# Hierarchical clustering analysis identifies metastatic colorectal cancers patients with more aggressive phenotype

**DOI:** 10.18632/oncotarget.21213

**Published:** 2017-09-23

**Authors:** Giuseppina Opinto, Nicola Silvestris, Matteo Centonze, Giusi Graziano, Rosamaria Pinto, Livia Fucci, Giovanni Simone, Anita Mangia

**Affiliations:** ^1^ Functional Biomorphology Laboratory, IRCCS-Istituto Tumori, Bari 70124, Italy; ^2^ Medical Oncology, IRCCS-Istituto Tumori, Bari 70124, Italy; ^3^ Scientific Direction, IRCCS-Istituto Tumori, Bari 70124, Italy; ^4^ Molecular Genetics Laboratory, IRCCS-Istituto Tumori, Bari 70124, Italy; ^5^ Pathology Department, IRCCS-Istituto Tumori, Bari 70124, Italy

**Keywords:** metastatic colorectal cancer, unsupervised hierarchical clustering analyses, immunohistochemistry, TMA, biomarker expression

## Abstract

A large percentage of metastatic colorectal cancer (mCRC) patients presents metastasis at the time of diagnosis. In the last years, great efforts have been made in the treatment of these patients with the identification of different phenotypes playing a key role in the definition of new systemic therapies. Unsupervised hierarchical clustering analysis (HCA) was performed considering the clinicopathological characteristics of 51 mCRCs. Using immunohistochemistry on tissue microarrays, we assessed the expression of β-catenin, NHERF1, RASSF1A, TWIST1, HIF-1α proteins in tumors and paired liver metastases. We also analyzed RASSF1A methylation status on the samples of the same patients. HCA distinguished Group 1 and Group 2 characterized by different clinicopathological features. Group 1 was characterized by higher number of positive lymph nodes (*p*=0.0139), poorly differentiated grade (*p*<0.0001) and high extent of tumor spread (*p*=0.0053) showing a more aggressive phenotype compared to Group 2. In both Groups, we found a common “basal” condition with a higher level of nuclear TWIST1 (*p*<0.0001 and cytoplasmic β-catenin (*p*<0.0001) in tumors than in paired liver metastases. Furthermore, the Group 1 was also characterized by RASSF1A hypermethylation (*p*<0.0001) and nuclear HIF-1α overexpression (*p=*0.0354) in paired liver metastases than in tumors.

In conclusion, HCA identifies mCRC patients with a more aggressive phenotype. Moroever, our results support the important contribution to the progression of the disease of RASSF1A methylation and the oncogenic role of HIF-1α in these patients. These evidences, should provide relevant information concerning the biology of this tumor and, as a consequence, potential new systemic therapeutic approaches.

## INTRODUCTION

Colorectal cancer (CRC) represents the third most frequently diagnosed cancer in USA with 135,000 cases in 2016 and with 50.000 patients dying because of this disease [1]. In the era of precision medicine, new prognostic factors (such as RAS/BRAF mutations, HER2 expression, and MSI) enhanced the prognostic significance of tumor-node-metastasis (TNM) staging in order to create new treatment strategies for CRC. Many genetic alterations trigger complex multistep molecular pathways involved in CRC development and in metastatization. Within the activating mutation of Wnt pathway causes cytoplasmic and nucleus accumulations of β-catenin [2]. Also, mutation of Adenomatous Polyposis Coli (APC) complex in CRC inhibits degradation of β-catenin and promotes its nuclear localization. This protein binds nuclear partners and promotes transcription of target genes such as *Jun*, *c-Myc* and *CyclinD-1* involved in cellular activation [2].

β-catenin interacts through its C-terminal PSD-95/Disc-large/ZO-1(PDZ) domain with Na^+^/H^+^ exchanger regulatory factor 1 (NHERF1), a scaffold protein characterized by two PDZ modules and a carboxyl (C)-terminal ERM-binding region [3]. NHERF1 displays a tumor suppressor role when it is localized at the plasma membrane, while it acts as a tumor promoter when it is localized in the cytoplasm or in the nucleus [4].

Moreover, the RAS/mitogen-activated protein kinase (MAPK) pathway plays an important role in CRC development [5]. RAS-association domain family 1 (RASSF1) is a tumor suppressor protein, containing a Ras association domain able to bind both RAS in a GTP-dependent manner and mediate the apoptotic effects of oncogenic RAS [6].

RASSF1A presents frequent transcriptional inactivation in tumor cells due to inappropriate methylation promoter [7, 8]. Nevertheless, RASSF1A methylation represents an alternative mechanism of aberrant RAS signaling in CRC [9].

One of the major mechanisms inducing the dissemination of cancer cells from the sites of the primary tumors is the Epithelial Mesenchymal Transition (EMT) [10]. Twist family bHLH transcription factor 1 (TWIST1) promotes EMT through the downregulation of E-cadherin [11, 12] and is considered a poor prognosis marker [13] and of chemoresistance in CRC [14].

The expression of TWIST1 is also regulated by Hypoxia-inducible factor (HIF)-1α [15], a protein associated to hypoxic tissue areas [16] and related to proliferation, differentiation, and development of an aggressive tumor phenotype [16]. After stabilization of HIF-α, the transcription factors translocate in the nucleus and activate target genes involved in metabolic reprogramming, genetic instability and tumorigenesis [17].

In this scenario, the aim of this study is to determine whether combined clinicopathological features, the level of methylation and proteins expression should identify biologically distinct groups of mCRC patients with paired liver metastases.

Thus, *i)*we evaluated the clinicopathological features of mCRC through the unsupervised hierarchical clustering method and *ii)*we assessed the expression of five molecular markers (β-catenin, NHERF1, RASSF1A, TWIST1, HIF-1α) and RASSF1A status in tumors and paired liver metastases of mCRC samples.

## RESULTS

### Patients characteristics

Of the 51 patients identified in the database of the Pathology Department of our Institute, 31 were males and 20 were females, with a median age of 63 years (range 44-85). All patients had adenocarcinoma, the tumor site was the colon in 29 cases and the rectum in 22 patients. Twelve tumors were classified as moderately differentiated and 39 were classified as poorly differentiated. The pathological staging was T2 in 1 case, T3 in 33 cases and T4 in 17 cases. Regarding the lymph node metastases (N), 1 case was N0, 13 were N1, 34 were N2 and 3 cases were N3.

### Immunohistochemistry for β-catenin, NHERF1, RASSF1A, TWIST1 and HIF-1α markers in T and LM cases

**Figure 1:**
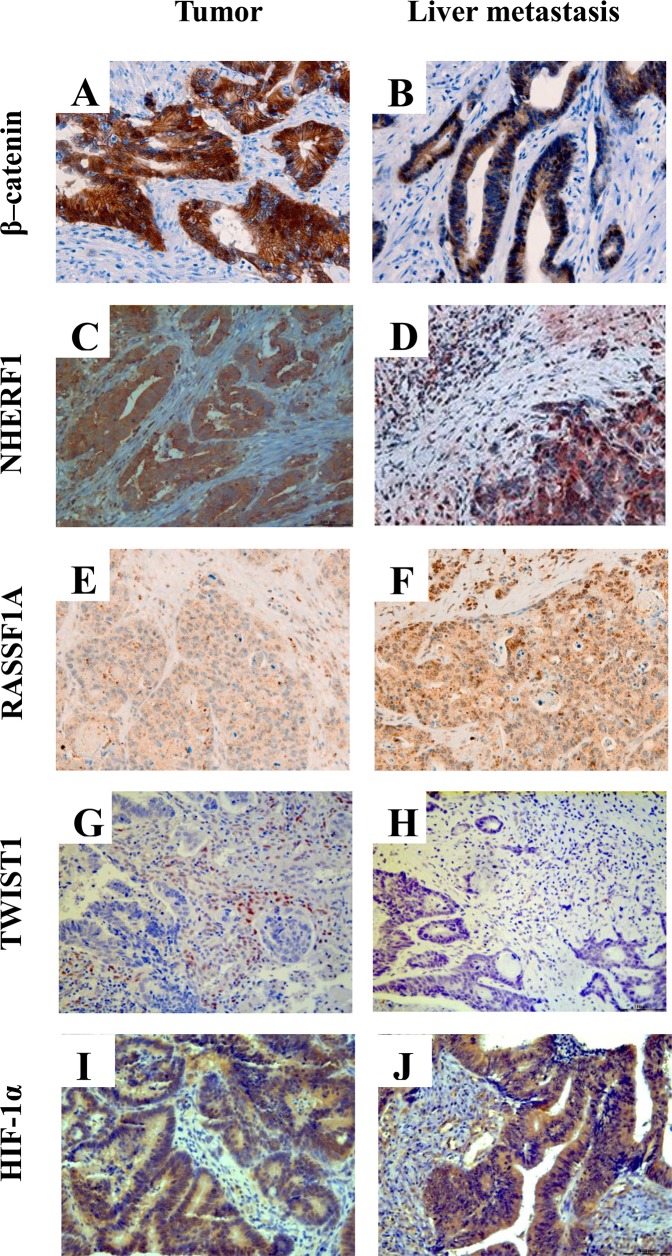
Representative images of immunohistochemical staining of β-catenin, NHERF1, RASSF1A, TWIST1 and HIF-1α proteins Membrane, cytoplasmic and nuclear localization of β-catenin in tumor **(A)** and in liver metastasis **(B)** NHERF1 immunostaining is present at the apical membrane, cytoplasm and nucleus in tumor **(C)** and in liver metastasis **(D)** Granular cytoplasmic staining of RASSF1A in tumor **(E)**, cytoplasmic and nuclear immunoreactivity of RASSF1A in liver metastasis **(F)** A nuclear staining of TWIST1 can be observed both in tumor **(G)** and in liver metastasis **(H)** Cytoplasmic and nuclear localization of HIF-1α in the cells of tumor **(I)** and liver metastasis **(J)** (original magnification x200).

Table 1 shows the different protein expression of β-catenin, NHERF1, RASSF1A, TWIST1 and HIF-1α, the staining localization, the range of expression and median value of positive cells in tumor (T) and paired liver metastasis (LM) tissues of the biomarkers. Representative images of the immunoreactivity of β-catenin, NHERF1, RASSF1A, TWIST1 and HIF-1α proteins in T and LM are shown in Figure 1.

**Table 1:** Expression of β-catenin, NHERF1, RASSF1A, TWIST1 and HIF-1α

Expression/localization		T		LM	
Biomarkers	Staining localization	Range%	Median%	Range%	Median%
**β-catenin**	Membranous	0-90%	75%	0-90%	50%
	Cytoplasmic	0-85%	30%	0-60%	0%
	Nuclear	0-50%	0%	0-28%	0%
**NHERF1**	Membranous	0-60%	5%	0-20%	0%
	Cytoplasmic	20-80%	60%	30-80%	70%
	Nuclear	0-68%	18%	0-72%	20%
**TWIST1**	Nuclear	0-100%	19%	0-31%	0%
**HIF-1α**	Cytoplasmic	0-90%	60%	0-95%	65%
	Nuclear	0-80%	0%	0-74%	0%
**RASSF1A**	Cytoplasmic	0-100%	30%	0-98%	60%

### RASSF1A methylation

We analyzed also the different RASSF1A promoter methylation in T and in paired LM. RASSF1A promoter methylation appeared significantly more frequently methylated in LM than in T (85% *vs*. 35%, *p*=0.015). In detail, the RASSF1A methylation median level was higher in LM than in the T compartment (15.33[3.03-48.69] and 0[0–0.67], respectively) in a statistically significant manner (*p* <0.0001, by Wilcoxon signed-rank test).

### Hierarchical cluster analysis

**Figure 2:**
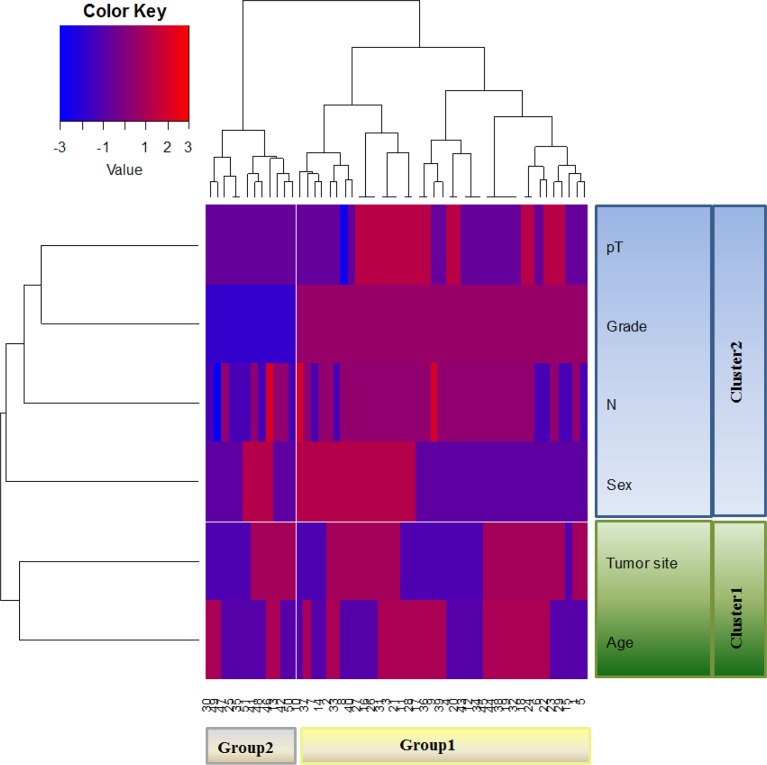
Unsupervised hierarchical analysis based on clinicopathological characteristics HCA identifies two distinct Groups (Group 1 and Group 2) of mCRC characterized by two clusters of different clinicopathological characteristics.

The hierarchical cluster analysis (HCA) was performed considering all the clinicopathological characteristics including age, tumor site, sex, N, tumor grade and depth of invasion (pT) of the 51 mCRC. The dendrogram (Figure 2) defined two sample groups (Group 1 and Group 2), characterized by two clusters of different clinicopathological characteristics (Cluster 1 and Cluster 2). In detail, Cluster 1 included age and tumor site, Cluster 2 included sex, N, tumor grade and pT of the patients. We analysed the distribution of each clinicopathological characteristic between Group 1 and Group 2 in order to identify which ones contributed to the formation of the two patient groups. We found statistically significant results only for N (*p*=0.0139), tumor grade (*p*<0.0001) and pT (*p*=0.0053), while the age, tumor site and sex were not significant. Group 1 was characterized by a higher number of positive lymph nodes, poorly differentiated grades and a high extent of tumor spread respect to Group 2. The Group 2 included patients with fewer positive lymph nodes, moderately differentiates grades and more limited tumor extension.

### Expression analysis of β-catenin, NHERF1, TWIST1, HIF-1α, and RASSF1A methylation analysis in Group 1 and Group 2 of metastatic CRC

In order to better define the features in Group 1 and Group 2 patients, in each group, we studied the different expression of markers between T and LM.

**Figure 3:**
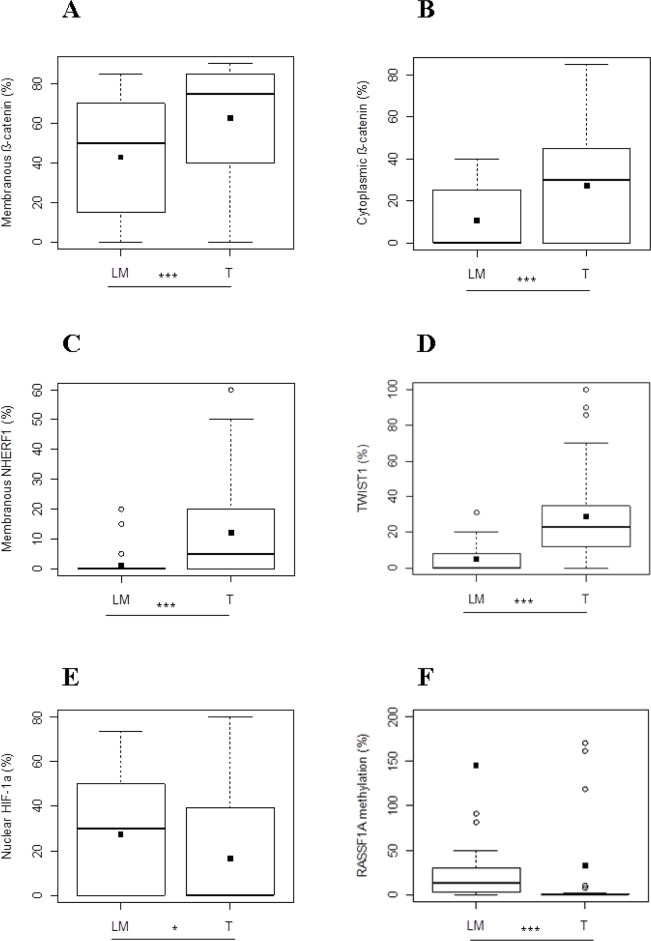
Protein expression analysis and RASSF1A methylation in metastatic CRC of Group 1 Different expression levels of membranous **(A)** and cytoplasmic β-catenin **(B)**, membranous NHERF1 **(C)**, TWIST1 **(D)**, nuclear HIF-1α **(E)** and RASSF1A methylation levels **(F)** between T and LM compartments. Black square in each box represents the mean percentage of positive cells. Circle indicates outlier and horizontal line in each box indicates the median. Abbreviation: T, tumor and LM, liver metastasis. ^***^p < 0.0001; ^**^p < 0.001; ^*^p < 0.05.

In Group 1, membranous β-catenin expression revealed a statistically significant difference between the two compartments and its median value was higher in T than in LM (75 [40-85] and 50 [15-70], respectively *p*<0.0001) (Figure 3A). Cytoplasmic β-catenin expression presented the same tendency and in particular its median expression was statistically higher in T than LM (30 [0-45] and 0 [0-25] respectively, *p*<0.0001) (Figure 3B). With respect to expression of membranous NHERF1, we noted that median value was statistically higher in T than in LM compartments (5 [0-20] and 0 [0-0], respectively *p*<0.0001) (Figure 3C). Protein analysis revealed that nuclear TWIST1 expression was differentially expressed between T and LM, in particular the median value of TWIST1 in T compartment was statistically higher than LM (23.38[11.04-37.01] and 0 [0-10] respectively, *p*<0.0001) (Figure 3D). Moreover, nuclear HIF-1α showed a significantly lower median value in T than LM (0 [0-42] and 30 [0-50]) respectively, *p*=0.0354) (Figure 3E).

Finally, the median value of the RASSF1A methylation level was significantly lower in T than LM (0[0-0.84] and 13.75[2.37-29.98], respectively (*p*<0.0001) (Figure 3F).

**Figure 4:**
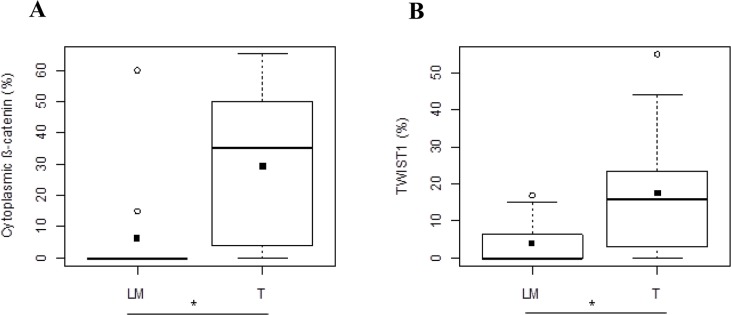
Protein expression analysis in metastatic CRC of Group 2 Different expression levels of cytoplasmic β-catenin. **(A)** and TWIST1 **(B)** between T and LM compartments. Black square in each box represents the mean percentage of positive cells. Circle indicates outlier and horizontal line in each box indicates the median. Abbreviation: T, tumor and LM, liver metastasis. ^***^p < 0.0001; ^**^p < 0.001; ^*^p < 0.05.

In Group 2, cytoplasmic β-catenin and nuclear TWIST1 expressions presented the same behavior observed in Group 1. In detail, cytoplasmic β-catenin expression showed a significantly different expression between T and LM (35 [4-50] and 0 [0-0], respectively) and in particular its median value was statistically higher in T than LM (*p*=0.0156) (Figure 4A). Moreover, we observed that nuclear TWIST1 was statistically higher in T than LM (15.91[3.25-23.71] and 0[0-6.50], respectively, *p*=0.0137) (Figure 4B).

### Correlation analysis among expression of β-catenin, NHERF1, RASSF1A, TWIST1, HIF-1α and the methylation status of RASSF1A in T and LM compartments of Group 1 and Group 2 of metastatic CRC

**Figure 5:**
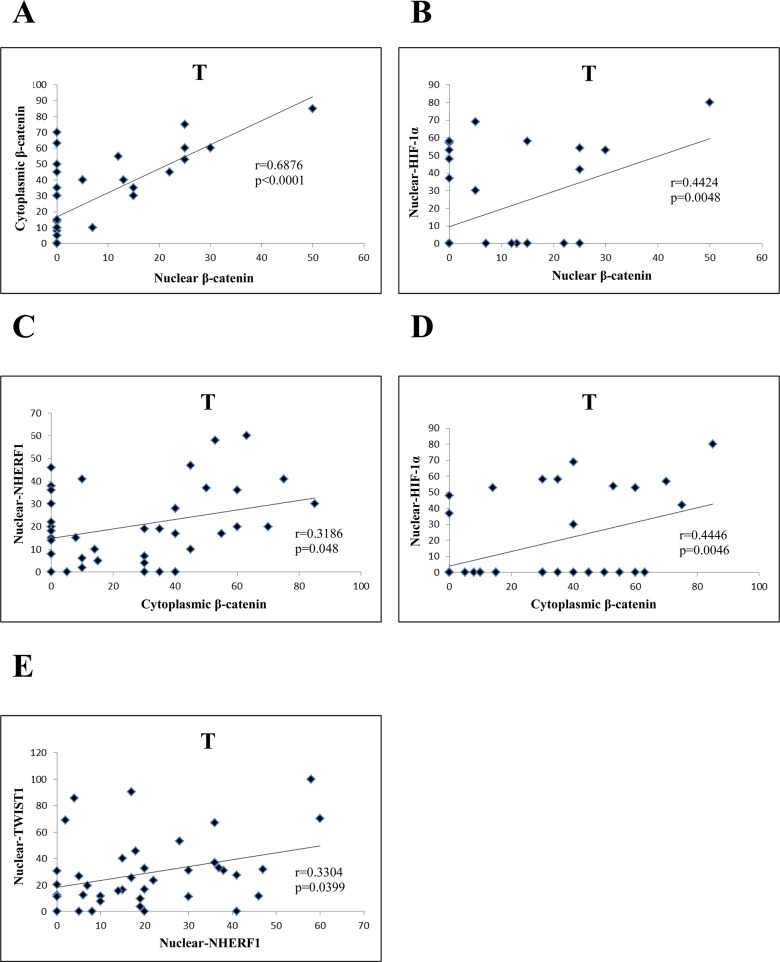
Correlation analysis among the expression of β-catenin, NHERF1, TWIST1 and HIF-1 α in T compartment of Group 1 Nuclear β-catenin correlated significantly with cytoplasmic β-catenin. **(A)** and nuclear HIF-1α **(B)** Cytoplasmic β-catenin was positively correlated to nuclear NHERF1 **(C)** and nuclear HIF-1α **(D)** NuclearNHERF1 correlated significantly with nuclear TWIST1 **(E)** Abbreviation: T, tumor.

Regarding the T compartment of Group 1, nuclear β-catenin was positively correlated to cytoplasmic β-catenin (*r=*0.6876*, p*<0.0001) (Figure 5A) and to nuclear HIF-1α (*r*=0.4424*, p*=0.0048) (Figure 5B). Moreover, cytoplasmic β-catenin was positively correlated to nuclear NHERF1 (*r*=0.3186, *p*=0.048) (Figure 5C), and nuclear HIF-1α (*r*=0.4446, *p*=0.0046) (Figure 5D). Nuclear NHERF1 was positively correlated to nuclear TWIST1 (*r*=0.3304, *p*=0.0399) (Figure 5E).

**Figure 6:**
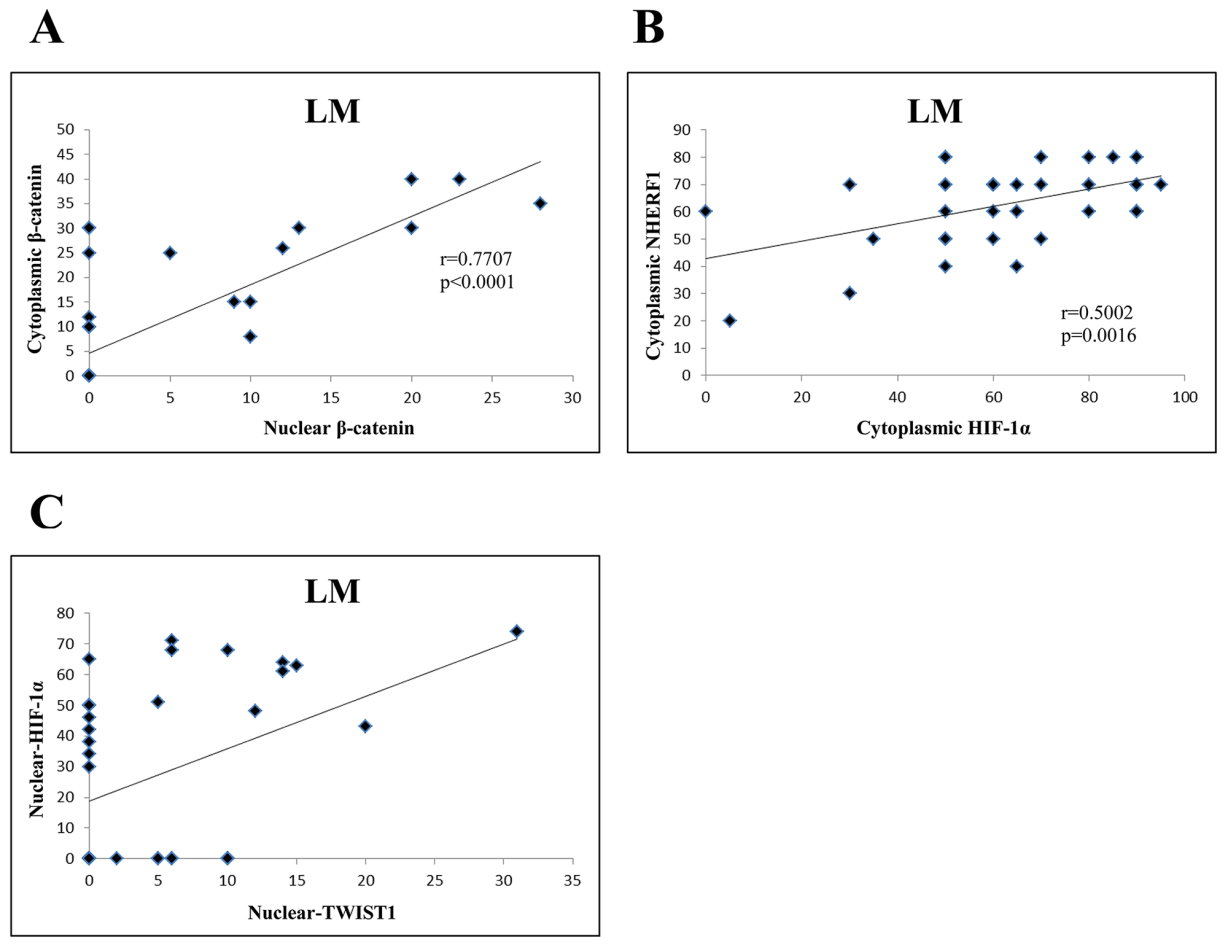
Correlation analysis among the expression of β-catenin, NHERF1, TWIST1 and HIF-1 α in LM compartment of Group 1 Nuclear β-catenin correlated significantly with cytoplasmic β-catenin. **(A)** Cytoplasmic HIF-1α was positively correlated to cytoplasmic NHERF1 **(B)** Nuclear TWIST1 correlated significantly with nuclear HIF-1α **(C)** Abbreviation: LM, liver metastasis.

Concerning LM compartment of the Group 1, there was a positive correlation between nuclear β-catenin and cytoplasmic β-catenin (*r=*0.7707, *p* < 0.0001) (Figure 6A), and between cytoplasmic NHERF1 and cytoplasmic HIF-1α (*r=0*.5002, *p*=0.0016) (Figure 6B). Moreover, nuclear TWIST1 was positively correlated to nuclear HIF-1α (*r*=0.4197, *p*<0.0097) (Figure 6C). In Group 2 the correlation analysis did not report statistically significant results in both T and LM compartments.

## DISCUSSION

Currently, tumor-node-metastasis staging remains the gold standard for prognostic classification of CRC. The American Joint Commission on cancer has recognized the growing need for more accurate and probabilistic individualized outcome prediction for precision medicine that would incorporate additional anatomic and non-anatomic prognostic factors beyond TNM [18].

In line with these requirements, we investigated the clinicopathological features of mCRC patients. Although all our cases are classified mCRC based on TNM staging system, hierarchical clustering analysis distinguished two groups of patients characterized by different clinicopathological characteristics including age, tumor site, sex, lymph node status, tumor grade and depth of invasion.

Hierachical clustering analysis showed that the Group 1 of patients was characterized by higher number of positive lymph nodes, poorly differentiated grade and high extent of tumor spread showing an aggressive phenotype compared to Group 2. The Group 2 included patients with fewer positive lymph nodes, moderately differentiated grades and more limited tumor extension. Moreover, we evaluated the level of RASSF1A methylation and the expression of β-catenin, NHERF1, RASSF1A, TWIST1 and HIF-1α in relation to clinicopathological characteristics in the T and in LM of Group 1 and Group 2. In Group 1, we observed a higher level of RASSF1A methylation in paired liver metastases than in primary tumors. In accordance with previous data, changes in DNA methylation can play a key role in the metastatic process [19]. In particular, a crucial role for RASSF1A in CRC progression has been demonstrated [9, 20].

Regarding the expression levels of the molecular markers analyzed, our results showed significant correlations among the expression of the proteins. In Group 1 and Group 2, we found a common “basal” condition characterized by a higher level of nuclear TWIST1 and cytoplasmic β-catenin in tumors than in paired liver metastases. EMT process is induced by TWIST1 [14] and it is also characterized both by the loss of E-cadherin and the β-catenin relocalization [21]. EMT is considered an early step of tumor invasion and metastases [22], when there is a transformation from epithelial to mesenchymal phenotype on the tumor front. This process could justify the lower expression of TWIST1 and cytoplasmic β-catenin in paired liver metastases respect to the tumors of the two Groups. Moreover, several studies have found a functional link between TWIST and β-catenin in cancers [23-25]. These additional data might also justify the same behavior of TWIST1 and cytoplasmic β-catenin in tumors and paired liver metastases of our cases.

Furthermore, HCA identifies in the Group 1 patients with more aggressive phenotype. The Group 1 is characterized by different protein expression level and by correlation analysis among some protein studied. We know that cytoplasmic β-catenin can move into the nucleus to activate transcription of LEF-1/TCFs, DNA binding proteins [26]. In Group 1, in tumors and in paired liver metastases, we noted a positive correlation between cytoplasmic and nuclear β-catenin suggesting a possible shift of the protein from the cytoplasm to the nucleus. In the physiologic condition, β-catenin is localized mainly at the membrane in intestinal epithelial tissue. The protein NHERF1 is necessary for β-catenin stabilization at the plasma membrane [27]. In the same Group 1, we observed a lower expression of membranous β-catenin and membranous NHERF1 in paired liver metastasis than in the tumor samples. These data are in agreement both with the loss of plasma membrane β-catenin found at the invasive front [28] and with the loss of physiologic functions of NHERF1 during the CRC progression [29].

Furthermore, in the tumor compartment of the Group 1, we detected a positive correlation between nuclear NHERF1 and cytoplasmic β-catenin. As has already been shown previously, NHERF1 accumulation was found both in the cytoplasm [29] and in the nucleus in invasive CRC front [30]. Here, NHERF1 correlated with β-catenin [4] suggesting a functional interaction between these two proteins.

Moreover, in the same Group 1, where TWIST1 was overexpressed in the tumor site, we noted also a positive correlation between nuclear NHERF1 and TWIST1. This result confirmed our previous study, that described the molecular relationship between nuclear NHERF1 and TWIST1 in more aggressive phenotype [30]. Besides TWIST1 and β-catenin, other signaling pathways have been implied in the activation of EMT, such as, HIF-1α. Interestingly, in Group 1, a higher expression of nuclear HIF-1α in liver metastases than in the tumors was observed. This data could suggest the oncogenic role of HIF-1α [17] also in our mCRC samples. We know that there is a large group of HIF-1α interacting proteins [31]. In our study, we did not evaluate the interaction between HIF-1α and the proteins analyzed. However, significant correlations between HIF-1α and some proteins were found. In specific, in tumors of Group 1 we reported two significant positive correlations between nuclear HIF-1α and nuclear β-catenin and between nuclear HIF-1α and cytoplasmic β-catenin. These results, are in agreement with previous evidence which have shown the stabilization of cytoplasmic β-catenin and nuclear translocation of the same protein modulated by HIF-1α [32]. In fact, some Authors, reported a cross-talk between HIF signaling and canonical Wnt signaling factors during tumor growth and metastatic process [33, 34].

Furthermore, TWIST1 expression is regulated by HIF-1α, [35]. Our results, showed that the expression of HIF-1α correlated with TWIST1 expression in tumor site of the Group 1 patients. This data was also in agreement with a study performed by Hung et al [36]. The Authors showed a co-expression of HIF-1α and TWIST1 correlated with a significant worse prognosis, in NSCLC patients. Our previous study described an association between NHERF1 and HIF-1α in mCRC [30]. Similarly, we confirmed a positive association between cytoplasmic NHERF1 and cytoplasmic HIF-1α in liver metastases of the Group 1.

In conclusion, hierarchical clustering analysis distinguishes two groups of mCRC patients (Group 1 and Group 2), characterized by different clinicopathological characteristics. In both Groups, we observed a common “basal” condition characterized by the involvement of nuclear TWIST1 and cytoplasmic β-catenin. In all tumor and paired liver metastasis samples, we found the same proteins behavior, confirming the functional link between nuclear TWIST1 and cytoplasmic β-catenin. Moreover, HCA identified in the Group 1 patients with more aggressive phenotype. These were also characterized by RASSF1A hypermethylation and an overexpression of nuclear HIF-1α in paired liver metastases than in tumors. Our results, support the important contribution of RASSF1A methylation and the oncogenic role of HIF-1α to the progression of the disease. These evidences, should provide relevant information concerning the biology of this tumor and, as a consequence, potential new systemic therapeutic approaches.

## MATERIALS AND METHODS

### Patients

This retrospective and not consecutive study involved 51 patients with a diagnosis of mCRC. All patients underwent surgery at the IRCCS Istituto Tumori “Giovanni Paolo II” of Bari between 2008 and 2015. Tumors were graded and classified independently by two double-blinded experienced pathologists according to the World Health Organization criteria [37]. Institutional Review Board approval for the use of human tissue in this study was given by the Research Ethics Committee of the IRCCS Istituto Tumori “Giovanni Paolo II”.

### TMA construction and Immunohistochemistry

Tissue microarrays (TMAs) were assembled from formalin-fixed and paraffin-embedded (FFPE) tissues of tumors and correspondently paired liver metastases as previously described [38]. Briefly, three different regions of tumors were identified and marked on haematoxylin and eosin stained sections. Sections were matched to their corresponding paraffin blocks (donor blocks), and three tumor cores with a diameter of 1 mm were punched from these tumor regions of each donor block and precisely arrayed into a new recipient paraffin block (TMA block) using the Galileo Tissue MicroArrayer CK 4500 (Transgenomic). Each sample was arrayed in triplicate to minimize tissue loss and to overcome tumor heterogeneity. The three cores were representative of the whole tumor sample. Four μm-thick slices were cut from the TMA blocks and transferred to slides. The TMA slides were stained by using two methods: a manual procedure for the detection of β-catenin, NHERF1, TWIST1 and HIF-1α proteins and an automated procedure for RASSF1A protein expression.

For the manual procedure, the sections were deparaffinized in xylene and rehydrated in graded ethanol solutions. Antigen retrieval was performed by the 0.01 M citrate buffer (pH 6.0) at 98°C in a water bath from a minimum of 30 to a maximum of 45 minutes. After cooling, endogenous peroxidase activity was blocked by incubation in 0.3% H_2_O_2_ buffer solution for 10 min, later the slides were incubated with 1% bovine serum albumin (BSA) in 1X phosphate-buffered saline (PBS) for 30 min to block non-specific protein binding. The sections were then incubated with primary antibodies against β-catenin (rabbit monoclonal anti-beta catenin antibody, E247, Abcam, Cambridge, UK; dilution 1:150), NHERF1 (rabbit polyclonal EBP50 antibody, PA1-090, Affinity Bioreagents, Golden, CO; dilution 1:150), TWIST1 (mouse monoclonal Abcam, Cambridge, UK, TWIST2C1a, 1:50) and HIF-1α (mouse polyclonal HIF-1alpha67, Abcam, Cambridge, UK, 1:100 dilution) overnight at 4°C.

According to the manufacturer's instructions the sections were incubated with anti-rabbit or anti-mouse secondary antibody conjugated with peroxidase labeled polymer (EnVision™+ System- HRP Labelled Polymer Anti-Rabbit or Anti-Mouse secondary antibody, Dako, CA, USA) for 1 hour at room temperature. The immunoreactivity of antibody was visualized by incubating the sections in 3-amino-9-ethylcarbazole (AEC + Substrate Chromogen, Dako, Carpinteria, CA, USA) for 15 minutes, except for anti β-catenin which requires the use of 3,3′-diaminobenzidine (Liquid DAB + Substrate Chromogen System, Dako, Carpinteria, CA, USA) for 8–10 minutes.

For the automated staining method, the sections were stained by using an automated procedure on the Benchmark XT platform (BenchMark XT, Ventana Medical Systems, Tucson, AZ). The slides were pretreated with Cell Conditioning1® for antigen unmasking and followed by pre-primary antibody peroxidase inhibition then incubated with the primary antibody incubation (mouse monoclonal anti-RASSF1a antibody, 3F3, Abcam, Cambridge, UK; dilution 1:30) for 1 h at 37°C.

The OptiView DAB IHC Detection Kit and OptiView Amplification Kit (Ventana Medical Systems) were used to detect RASSF1A protein expression. Finally, tissues were counterstained with Haematoxylin II and Bluing Reagent, then were dehydrated and mounted.

Positive and negative controls were included in each staining run as indicated in the data sheet of each antibody. For negative control, the primary antibody was omitted and replaced by PBS 1X pH 7.6. The accuracy, reliability and reproducibility of these antibodies (β-catenin, NHERF1, TWIST1, HIF-1α and RASSF1A) have been validated in previous studies already published [19, 30, 39, 40].

Protein expression was quantified by counting the positive cells in each core on TMA at x20 magnification and expressed as a percentage of positive cells/core. Only immunostaining of invasive cancer cells within the tissue cores were considered. The mean of three readings relative to the three cores for each tumor sample was calculated and represented the protein expression of each tumor. If one core was uninformative, or either lost or contained no tumor tissue, the overall score applied was that of the remaining cores. Furthermore, the cases in which all three cores were uninformative were considered non-assessable and excluded from the analyses.

The cores were independently evaluated for β-catenin, NHERF1, RASSF1A, TWIST1 and HIF-1α expression in T and LM tissues by two observers blind to patient outcome and clinicopathological data. Any discrepancies between the two observers were resolved by re-examination and consensus.

The immunoreactivity of β-catenin and NHERF1 proteins were evaluated separately for the cell membrane, cytoplasmic and nuclear compartments. For TWIST1 only nuclear localization was considered, the immunoreactivity of HIF-1α was examined separately for cytoplasmic and nuclear localization.

RASSF1A immuno-expression was assessed as the percentage of labeled cytoplasmic and in few cases was observed as mixed cytoplasmic/nuclear staining where it was present [41].

### DNA extraction and quantitative methyl-specific PCR (QMSP) analysis

DNA was extracted by T and LM tissues containing more than 70% of cancer cells. Samples were digested with SDS/proteinase K over night at 56°C, and DNA was extracted with the QIAamp DNA FFPE Tissue kit (Qiagen, Valencia CA) according to the manufacturer's protocol. Concentrations were estimated with the ND-8000 Spectrophotometer (NanoDrop Technologies, Wilmington, DE).

DNA extracted from T and LM specimens were subjected to bisulphite treatment and DNA purification using the Epitect Bisulfite kit (Qiagen, Valencia CA) according to the manufacturer's instructions. The modified DNA was used as a template for real-time fluorogenic MSP. Amplification reactions were carried out in triplicate, primers and probe of the gene of interest were designed to specifically amplify the bisulphite modified region containing the putative methylated CpGs, whereas the primer and probe for the reference gene (ACTB) were designed to specifically amplify a bisulphite modified region not containing CpGs. Amplification reactions were carried out in 96-well plates on a 7000 Sequence detector (ThermoFisher Scientific). Each plate included patient DNA samples, positive (CpG Genome Universal Methylated DNA, a completely methylated DNA) and negative (normal leukocyte DNA or DNA from a known unmethylated cell line) controls, and multiple water blanks. Serial dilutions (90-0.009 ng) of CpG Genome Universal Methylated DNA were used to construct a calibration curve for the ACTB gene and for the gene of interest. The relative level of methylated DNA in each sample was determined as a ratio of the quantity mean of the target gene to the quantity mean of the ACTB and then multiplied by 1,000 for easier tabulation [(average value of triplicates of target gene/average value of triplicates of ACTB) × 1,000]. A series of 10 normal colon tissues were used as a calibrator and presented a QMSP level of the considered gene < 1. As a consequence, a gene was considered “methylated” when the QMSP level was ≥1.

### Statistical methods

HCA was performed using the value of age, sex, tumor site, N, grade and pT, clinicopathological characteristics, in order to identify subgroups of patients with similar profiles. The variables have been defined according to the clinical standards, while for age the median value (63 years) has been chosen as cut off. The Ward agglomeration method was used to obtain clusters both for clinicopathological characteristics and cases, computing the distance matrix with the Euclidean distance. A heatmap was produced to facilitate the interpretation: red blocks represent a positive score, blue blocks represent a negative score. The Pearson's Chi-square Test, or Fisher's Exact test when appropriate, was assessed to determine which characteristic contributed to the identification of two main cluster groups. Comparisons of the expression levels of the biomarkers between tissues (tumoral and metastatic) were assessed with the Wilcoxon signed-rank test, separately for Group 1 and Group 2. The correlation between the biomarkers was also considered and calculated using Pearson's Correlation Coefficient (r). Statistical significance was achieved at a p-value (p)<0.05. All the analyses were performed using the Statistical Analyses Software (SAS, Release 9.4, Cary, NC, USA).

## Abbreviations

APC: adenomatous polyposis coli
BSA: bovine serum albumin
CRC: colorectal cancer
EMT: epithelial-mesenchymal-transition
FFPE: formalin-fixed and paraffin-embedded
HCA: hierarchical clustering analyses
HIF-1α: hypoxia-inducible factor-1α
LM: paired liver metastases
MAPK: mitogen-activated protein kinase
N: lymph node metastases
NHERF1: Na^+^/H^+^ Exchanger Regulatory Factor 1
p: p-value
PBS: phosphate buffered saline
PDZ: PSD-95/Disc-large/ZO-1
pT: depth of invasion
r: correlation coefficient
RASSF1A: RAS-association domain family 1
SAS: statistical analyses software
T: tumor
TNM: tumor-node-metatstasis
TMAs: tissue microarrays
TWIST1: Twist family bHLH transcription factor 1
